# Enzymatically Polymerized Organic Conductors on Model Lipid Membranes

**DOI:** 10.1021/acs.langmuir.3c00654

**Published:** 2023-06-02

**Authors:** Diana Priyadarshini, Chiara Musumeci, David Bliman, Tobias Abrahamsson, Caroline Lindholm, Mikhail Vagin, Xenofon Strakosas, Roger Olsson, Magnus Berggren, Jennifer Y. Gerasimov, Daniel T. Simon

**Affiliations:** † Laboratory of Organic Electronics, Department of Science and Technology, Linköping University, 601 74 Norrköping, Sweden; ‡ Department of Chemistry and Molecular Biology, University of Gothenburg, 412 96 Gothenburg, Sweden; § Chemical Biology and Therapeutics, Department of Experimental Medical Science, Lund University, 221 84 Lund, Sweden

## Abstract

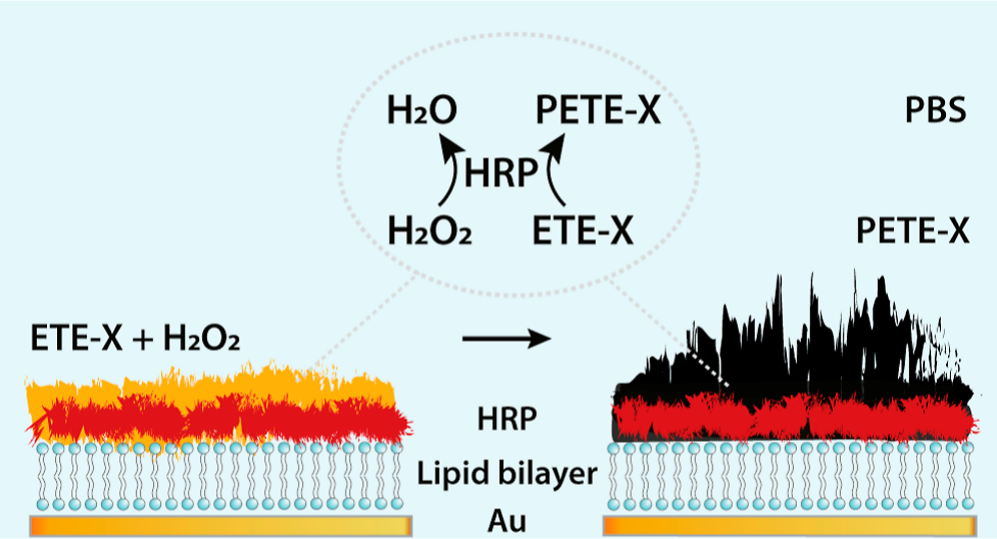

Seamless integration between biological systems and electrical components is essential for enabling a twinned biochemical–electrical recording and therapy approach to understand and combat neurological disorders. Employing bioelectronic systems made up of conjugated polymers, which have an innate ability to transport both electronic and ionic charges, provides the possibility of such integration. In particular, translating enzymatically polymerized conductive wires, recently demonstrated in plants and simple organism systems, into mammalian models, is of particular interest for the development of next-generation devices that can monitor and modulate neural signals. As a first step toward achieving this goal, enzyme-mediated polymerization of two thiophene-based monomers is demonstrated on a synthetic lipid bilayer supported on a Au surface. Microgravimetric studies of conducting films polymerized in situ provide insights into their interactions with a lipid bilayer model that mimics the cell membrane. Moreover, the resulting electrical and viscoelastic properties of these self-organizing conducting polymers suggest their potential as materials to form the basis for novel approaches to in vivo neural therapeutics.

## Introduction

Electrical devices for the recording or triggering of neural signals are of immense importance in the study of neurodegenerative disorders and fundamental neuroscience. In recent years, conducting polymer electrodes have overcome many limitations of rigid metal predecessors in bridging the technology–biology gap by matching their elasticity to that of the nervous system and reducing the impedance of the interface. However, despite being flexible and electrically matched, these polymer electrodes are generally patterned on two-dimensional substrates, which limits their usefulness in probing the three-dimensional space of the nervous system.^1−3^

One way of bypassing the limitations of electrodes patterned on a plane is to use existing biological structures as the three-dimensional substrate around which conducting polymer electrodes deposit. Several approaches to inducing the polymerization of monomer precursors of conducting polymers have thus far been implemented. In a foundational study, electropolymerization was used to form polyethylene dioxythiophene (PEDOT)-based conductors around the structures of cells and neural tissue.^4,5^ The development of new materials with a lower oxidative potential has now made it possible to eschew external stimuli altogether in favor of polymerization by oxidative enzymes. A novel conjugated polymer based on a monomer with a 2,5-bis(2,3-dihydrothieno[3,4-b][1,4]dioxin-5-yl)thiophene (ETE) backbone that has been functionalized on the central thiophene with an ethoxy-1-butanesulfonic acid side chain (ETE-S) has already been used to fabricate electrodes, capacitors, and transistors in vivo by taking advantage of the innate oxidative defense pathways to induce polymerization in several plant species and in the small aquatic animal *Hydra vulgaris*(6−8) and most recently in living fish brains and medicinal leeches.^9^ Recently, the electroactive polymer polyaniline (PANI) was locally synthesized in the presence of hydrogen peroxide (H_2_O_2_) in rat hippocampal neurons and worm pharyngeal muscle cells that were genetically modified to express ascorbate peroxidase (Apex-2) enzyme.^10^ Materials polymerized enzymatically in vivo are a promising pathway for bioelectronics since they allow for selective interfacing with specific cells or cell types, in addition to being soft and having improved impedance matching.^9^

Despite several studies establishing the feasibility of organizing conducting polymers around living tissue, it remains unclear how polymers and monomer precursors interact with lipid bilayers that form the basis of the cell membrane or how enzymatic oxidative polymerization affects the structure of lipid bilayers. Electrochemical quartz crystal microbalance with dissipation (EQCM-D) with in situ electrochemical impedance spectroscopy (EIS) is proposed to be a powerful surface characterization technique to assess the processes occurring at the cell surface during polymerization because it provides information on both structural and electrical film properties. EQCM-D can then be used to effectively screen potential monomer candidates and diagnose the cause of failed polymerization. As a model system in validating this technique, the polymerization of ETE-based derivatives by the enzyme horseradish peroxidase (HRP) adsorbed on a cationic-supported lipid bilayer (SLB) of 1,2-dioleoyl-3-trimethylammonium-propane (DOTAP) in a phosphate buffered saline (PBS) medium is evaluated. To probe a range of possible interactions, the polymerization of two differently functionalized ETE derivatives—an anionic sulfonate-modified derivative (ETE-S) and a zwitterionic phosphocholine-modified derivative (ETE-PC)—is explored as it has been shown that side-chain functional groups play a significant role in polymer–substrate interactions.^11^ Using EQCM-D, it was possible to identify differences in the activity of HRP, as well as in the interactions between the resulting poly(ETE-S) (PETE-S) or poly(ETE-PC) (PETE-PC) polymers and the Au/bilayer surface. To date, this is the first demonstration of in situ formation of a conducting polymer on a lipid bilayer.

## Materials and Methods

### Chemicals and Reagents

ETE-S and ETE-PC were synthesized as per published protocols.^12,13^ DOTAP, 10 mg/mL in CHCl_3_, was purchased from Avanti Polar Lipids. A 35 wt % H_2_O_2_ solution in H_2_O was purchased from Acros Organics; HRP Type I and PBS tablets were purchased from Sigma-Aldrich. Ammonium hydroxide (25% solution) was purchased from EMSURE. Enhanced K-Blue solution containing both 3,3′,5,5′ tetramethylbenzidine (TMB) and H_2_O_2_ as well as a 1N H_2_SO_4_ stop solution was purchased from NEOGEN. Stock solutions were prepared at a concentration of 1 mg/mL (monomer, lipids, and HRP) or 1 M (H_2_O_2_) in PBS (137 mM NaCl, 2.7 mM KCl, and 10 mM phosphate buffer, pH 7.4 at 25 °C) and refrigerated for the week, from which the required diluted aliquots at 0.1 mg/mL in PBS at room temperature were freshly prepared on each measurement day.

### Vesicle Preparation

A 1 mg/mL stock solution of DOTAP vesicles having a 100 nm diameter in PBS was prepared by completely evaporating CHCl_3_ from 0.1 mg/mL of the original lipid solution using a gentle N_2_ gas stream, resuspending the residue in PBS by stirring the mixture on a magnetic stir plate for 1 h, and then extruding the resulting solution 11 times using a mini extruder kit (Avanti) with a pore size of a 0.1 μm (Whatman Nuclepore). The solution was refrigerated and consumed within a week.

### In Situ Polymerization

Au–Ti quartz sensors (5 MHz, Biolin Scientific) were used for the EQCM-D measurements. Sensors were cleaned based on the manufacturer’s recommended protocol by incubating them in a TL1 cleaning solution (DI water, 31% H_2_O_2_, and 25% NH_4_OH mixed in a ratio of 5:1:1) for 7 min at 100 °C. Sensors were then rinsed with DI water and dried under a gentle stream of N_2_ gas and placed in a UV ozone cleaner (Novascan Technologies) for 30 min. Simultaneous QCM-D and electrochemical measurements were obtained using QSense E4 Analyzer (Biolin Scientific) coupled to an IPC high-precision multichannel pump (ISMATEC) and μAutolab III potentiostat (Metrohm). EIS recordings were obtained using the Au–Ti sensor as the working electrode, the Pt plate of the EQCM-D module as the counter electrode, and an external Dri-Ref Ag/AgCl reference electrode (World Precision Instruments). QCM-D was measured at 22 °C and a flow rate of 0.1 mL per minute. A stable baseline was established in PBS for ∼15 min prior to the start of each QCM-D experiment. Each solution was allowed to flow until the entire 1 mL was fully consumed, after which PBS was reintroduced to rinse off unattached entities. In the case of ETE-S or ETE-PC mixed with H_2_O_2_, however, instead of washing with PBS immediately after the monomer solution was consumed, the flow was stopped for at least 30 min, or until the frequency drop stabilized, thereby allowing sufficient time for polymerization, after which PBS was reintroduced into the cell as usual to remove unattached residues. EIS measurements were taken during the PBS rinse. All EIS data were obtained at 0 V vs the open-circuit potential, using an AC sine wave input signal with an amplitude of 10 mV and frequency scanned from 1 MHz to 0.1 Hz at the distribution of 7 points per decade. Results were analyzed using QSoft, QTools, and QSense Dfind (for QCM-D data) and Nova 2.1 (for EIS data) software.

### AFM

Sensors that were used in the QCM-D experiments were dried within the flow modules by pumping air for ∼5 min, after which they were taken out, lightly dabbed near the edges with a tissue paper to remove excess water, and set aside to dry completely under ambient conditions. The films formed on each sensor were then observed using a Dimension 3100 AFM (Veeco Digital Instruments, Bruker) and scanned in tapping mode using Si probes (BudgetSensors) having a force constant of 40 N/m to obtain corresponding height and phase images. Basic image clean-up operations like leveling the data by mean plane subtraction, aligning the rows using the median method, and correcting horizontal scars or strokes were performed using the image capture and processing software: Nanoscope v.531r1 for image acquisition and Gwyddion 2.46 for image processing.

### HRP Assay

The activity of the immobilized HRP enzyme was examined on bare Au and DOTAP bilayer-modified QCM-D sensors using the Enhanced K-Blue TMB substrate according to the standard procedure.^14−16^ The deposition of HRP was followed by a PBS rinse in the QCM-D flow modules to remove the weakly adsorbed enzyme, after which sensors were placed in separate vials containing 1 mL of PBS. To each of these vials, 1 mL of the chromogenic TMB substrate was added and the samples were allowed to incubate for 30 min under ambient conditions. The UV–vis spectra were acquired using a Synergy H1 microplate reader (Bio-Tek) by transferring 100 μL of each of the vial contents into a well of a polypropylene 96-well F-bottom clear microplate (Greiner Bio-One) containing 100 μL of a 1N H_2_SO_4_ solution to stop the enzyme–substrate reactions. The absorbance was quantified at a wavelength of 450 nm. Blank solution wells consisting of 50 μL each of PBS and TMB, along with 100 μL of 1 M H_2_SO_4_ to match the volume level and mix of sample solution wells, were also measured for reference.

### Ex Situ Polymerization

An additional control experiment was conducted using a microplate reader to evaluate the effect of charged DOTAP lipids on the doping state of the enzymatically polymerized ETE-S and ETE-PC. Equal volumes of the inflow samples used for QCM-D measurements (i.e., 200 μL of each of HRP at 0.1 mg/mL, DOTAP at 0.1 mg/mL, and ETE-X at 0.1 mg/mL with H_2_O_2_ at 1 mM) were mixed in four 1.5 mL Eppendorf tubes and set aside for 30 min to obtain PETE-S and PETE-PC in the presence and absence of DOTAP lipids. Spectrophotometric analyses were then conducted as described in the previous section, by measuring the average absorbance spectra from 300 to 999 nm at the rate of 1 nm per step, for 100 μL of each sample, with three wells per sample.

## Results and Discussion

**Figure 1:**
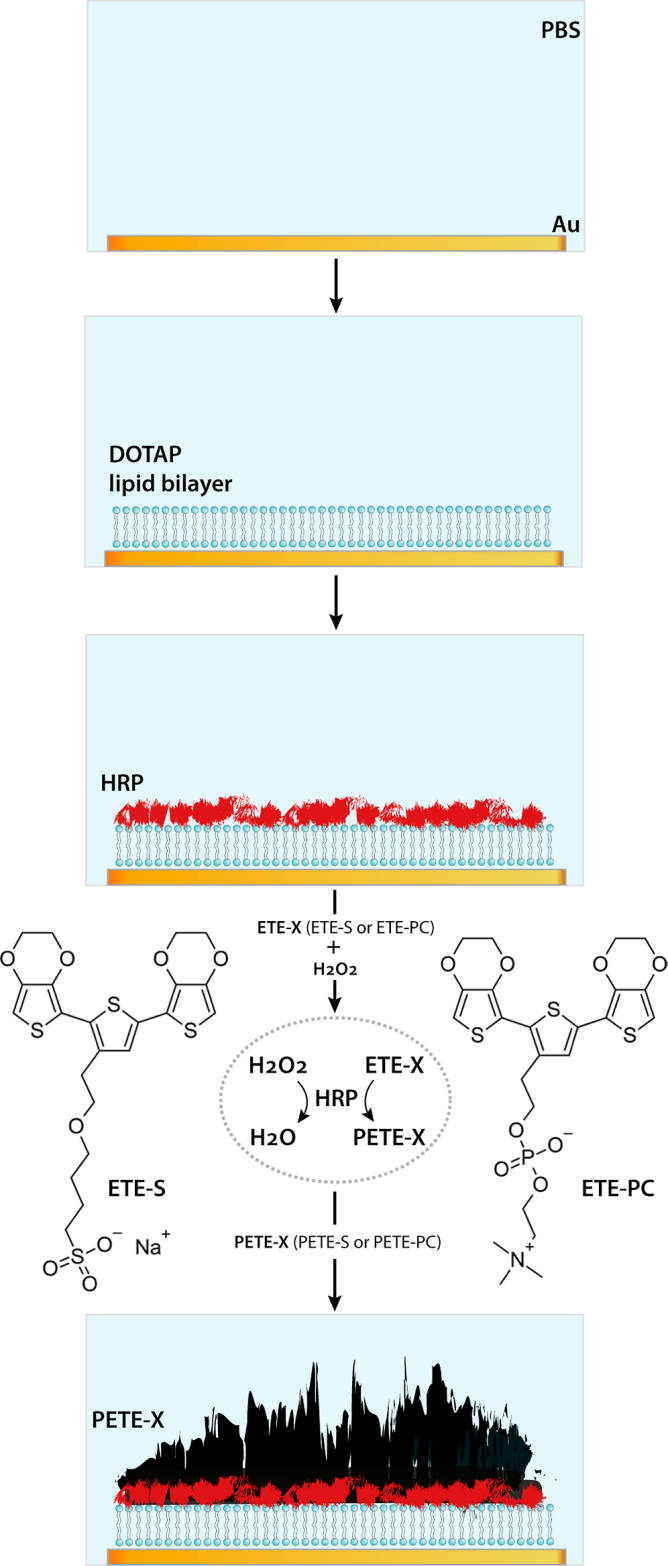
Schematic representation of enzymatic in situ polymerization of ETE-X, i.e., ETE-S or ETE-PC, on a DOTAP lipid bilayer supported on a Au substrate, resulting in PETE-X (PETE-S or PETE-PC). Polymerization occurred in PBS and was catalyzed by HRP, which was deposited on the substrate prior to monomer introduction. Chemical structures of the anionic ETE-S and zwitterionic ETE-PC are also shown.

QCM-D and EIS were used to characterize the adsorption processes on a gold-coated sensor upon the sequential introduction of (1) DOTAP vesicles, (2) HRP, and (3) an aqueous solution of the monomer precursor and hydrogen peroxide which gets catalyzed by the adsorbed HRP to form a conducting polymer film on the sensor surface (Figure 1). The enzyme HRP has a broad selectivity for substrates and is shown to polymerize ETE-S in the presence of H_2_O_2_ which acts as the oxidant.^6−8^ Real-time QCM-D measurements were complemented by representative EIS measurements at the end of each stage of the process. A buffer rinse was performed after each mass adsorption stage until associated frequency and dissipation shifts stabilized, at which point the EIS measurement was recorded. As controls, the surface-confined processes in the absence of an SLB and in the absence of H_2_O_2_ were also evaluated. Additionally, QCM-D samples were imaged using atomic force microscopy (AFM) to obtain topographical information about the resulting polymers.

### SLB Formation

**Figure 2:**
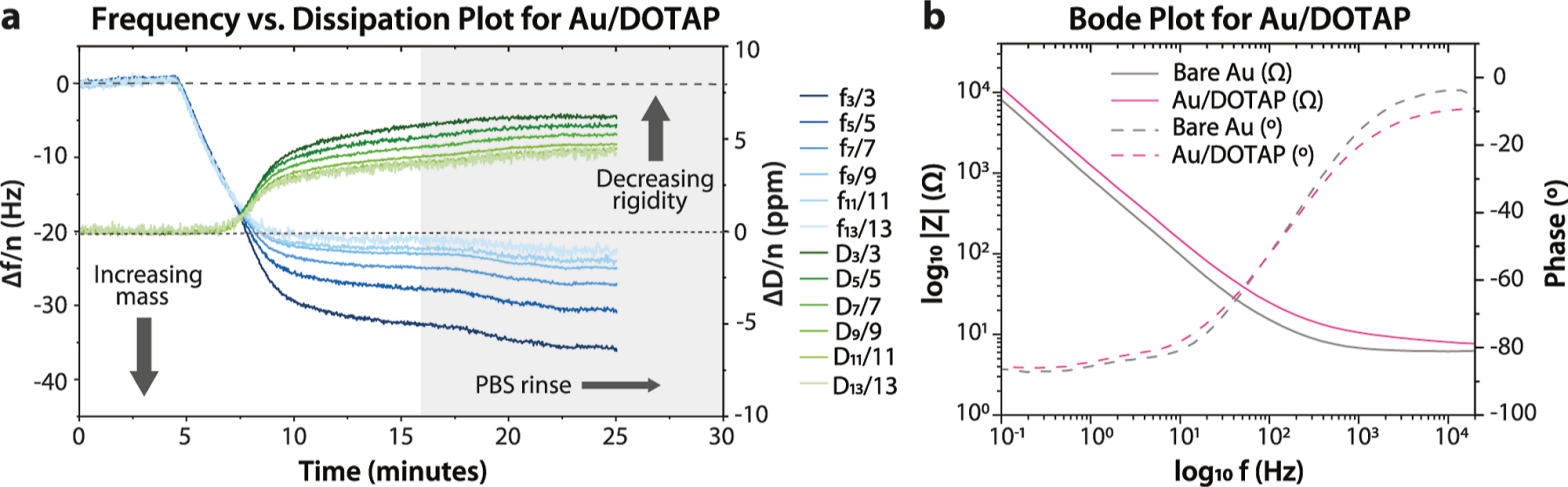
(a) QCM-D and (b) EIS responses to the formation of a DOTAP bilayer on a gold substrate. In the QCM-D plot (a), the horizontal dashed line indicates the zero level for frequencies, while the horizontal dotted line indicates the zero level for dissipation values. Layer mass increase occurs with increasing negative frequency shifts (downward), while the layer becomes less rigid with increasing positive dissipation shifts (upward). The gray shaded region indicates the PBS rinse phase. Both the frequency and dissipation shifts are normalized to their respective overtone numbers.

Upon introduction of the DOTAP vesicle solution to a bare gold surface, the unilamellar DOTAP vesicles readily ruptured and rearranged to form a stable lipid bilayer on the sensor surface as indicated in Figure 2a. Shifts in the frequency are proportional to changes in the hydrated mass of the adsorbed film, while changes in the dissipation are related to the viscoelastic properties of the film. A drop in the frequency (i.e., more negative frequency relative to a reference point at the beginning of the measurement) is associated with an increase in the adsorbed mass, whereas an increase in the dissipation (i.e., more positive dissipation) is associated with an increase in film softness. Furthermore, information about these real-time changes across the entire thickness of the film can be obtained by observing the overtone distribution since signal penetration depth decreases with increasing overtone number.^17^ Frequency change in the range of −27 Hz was noted to confirm the SLB formation due to lipid vesicle rupture, although slightly higher than the expected 0 range for the dissipation values could indicate the presence of partially ruptured lipid vesicles.^18−20^ EIS spectra, acquired before and after the introduction of DOTAP vesicles (Figure 2b), show an increase in impedance upon bilayer formation owing to the increased distance between the Au surface and the ions in the electrolyte forming the capacitor of the electrical double layer. This increase is particularly prominent in the midfrequency range, which is typically associated with interactions of the bilayer membrane with electrodes and electrolytes.^21−23^

### HRP Deposition

**Figure 3:**
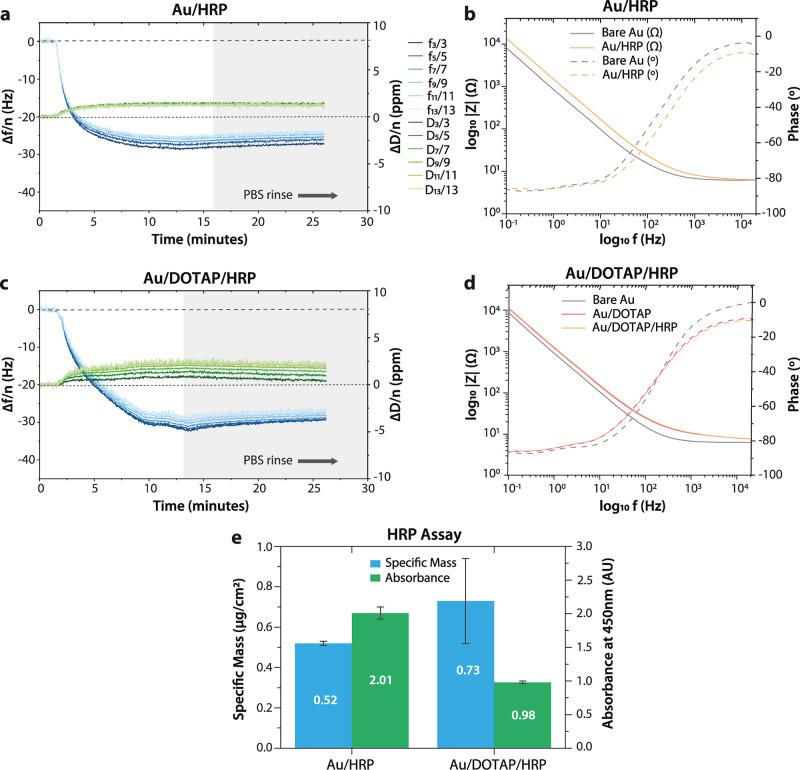
(a,c) HRP deposition QCM-D measurements (overtone-normalized) and (b,d) in situ EIS measurements acquired upon the adsorption of HRP on bare gold and DOTAP-modified QCM-D sensors. Results of the (e) enzyme activity assay are also presented along with the specific mass values estimated using Kelvin-Voigt viscoelastic modeling of the QCM-D data; error bars correspond to standard errors calculated from at least three replicates.

The enzyme HRP, with the ability to catalyze the oxidation of a broad range of substrates including ETE derivatives, was introduced to the QCM-D flow module at a concentration of 0.1 mg/mL. The amount of adsorbed mass, effects on the electrical characteristics of the interface, and the activity of the adsorbed enzyme were evaluated for the enzyme deposited on both bare gold substrates and DOTAP-modified substrates (Figure 3). The total adsorbed mass, as quantified by QCM-D, does not vary significantly (*P*-value 0.3696, which is ≫0.05)^24^ between the bare gold (0.52 ± 0.01 μg/cm^2^) substrate and the DOTAP layer (0.73 ± 0.21 μg/cm^2^) and is within the range of what was observed for a monolayer of HRP deposited within a phospholipid support.^25^ However, considering that the average activity of HRP on Au samples is 105% more than that for HRP on Au/DOTAP samples, HRP is inferred to be more active in the absence of DOTAP than in its presence. In the EIS spectra, HRP adsorption causes an observable change in the impedance of the interface on gold substrates while very little change is apparent when HRP is adsorbed on the DOTAP-modified surface despite the similar quantity of adsorbed HRP.

### Enzymatic Polymerization

**Figure 4:**
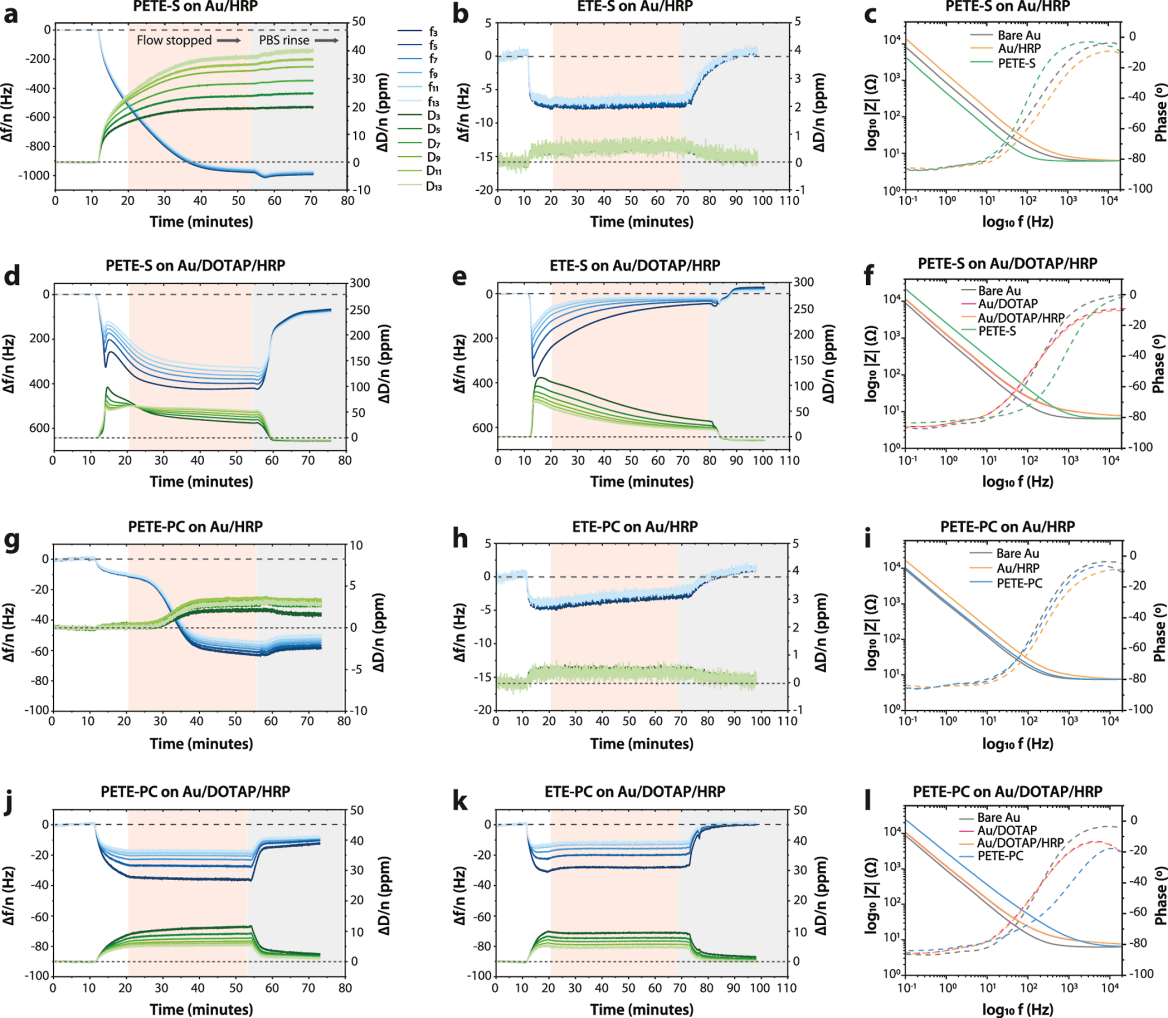
Overtone-normalized QCM-D measurements during (a,d,g,j) polymerization and (b,e,h,k) adsorption of ETE-S and ETE-PC on different substrates (bare and DOTAP-modified gold). The white region contains the time point where the monomer was introduced under constant flow, the pink region indicates where the flow was stopped, and the gray region indicates where the surface was rinsed with PBS buffer. Horizontal zero-level lines correspond to the level of HRP adsorption on Au or Au/DOTAP substrates. (c,f,i,l) The EIS data are reported at each stage of surface modification for the measurements involving polymers.

Enzymatic polymerization of ETE-S and ETE-PC, catalyzed by immobilized HRP, was monitored in situ on bare Au substrates, as well as on the DOTAP bilayer using EQCM-D (Figure 4). A freshly made solution containing 0.1 mg/mL ETE-X monomer and 1 mM H_2_O_2_ was introduced to induce polymerization on the QCM-D sensor surface. To isolate the effects of polymerization from the effects of monomer interaction with the surface, monomer adsorption in the absence of H_2_O_2_ was also monitored independently using EQCM-D. The frequency and dissipation responses were measured continuously over the course of the polymerization, while the EIS was measured before and after polymer formation.

As before, a drop in the QCM frequency is associated with an increase in the adsorbed mass, whereas an increase in the dissipation is associated with an increase in film softness. QCM-D data indicate that enzymatic polymerization is initiated as soon as the H_2_O_2_-monomer solution reaches the HRP-modified substrate, resulting in a mass increase that reaches equilibrium within 40 min. Unless otherwise noted, the adsorbed mass was quantified (Table S1) using Kelvin–Voigt viscoelastic modeling based on reported lipid bilayer experiments investigating rigid or soft films.^17,26^ Additional viscoelastic properties like shear moduli^27^ of the polymers (Table S1) were converted to Young’s moduli by multiplying corresponding values by 2.7, with the assumptions of the material being isotropic and having a Poisson ratio of 0.4.^28,29^ The resulting Young modulus for the polymer on Au/HRP substrate was 5.1 MPa for PETE-S and 1.0 MPa for PETE-PC. These estimated values are lower than the reported 10 MPa–3 GPa range of other solid or thin-film conjugated polymers and closer to the ∼0.01 MPa of biotic living tissue and ∼0.6 MPa of human skin.^30,31^

On bare gold, PETE-S forms a layer that gradually increases in thickness to 16.5 μg/cm^2^ and adheres well to the underlying substrate. In the absence of H_2_O_2_, the ETE-S monomer adsorbs to gold reversibly and is fully removed during the PBS rinse. EIS shows that the deposition of PETE-S significantly reduces the impedance of the HRP-modified gold surface, indicating that the polymer forms a good electrical contact with Au. In contrast, PETE-S deposition on the DOTAP bilayer displays evidence of several processes occurring simultaneously. To extract the effects of each of these processes, it is helpful to first examine the monomer interaction with the DOTAP bilayer. When the ETE-S monomer is introduced at a DOTAP bilayer, there is a sharp initial reduction in frequency accompanied by a large increase in dissipation, after which the frequency and dissipation both decrease in magnitude and slowly equilibrate to low values. During the PBS rinse, the frequency shift equilibrates beyond the baseline HRP levels (horizontal zero-level lines). This behavior reveals that the anionic ETE-S monomer acts as a surfactant that first forms soluble complexes with the DOTAP lipids, which are then easily washed away from the surface during the PBS rinse. The formation of the PETE-S polymer on the DOTAP bilayer exhibits the signatures of both polymer deposition (as for PETE-S on gold) and bilayer disruption (as for ETE-S on DOTAP). While bilayer disruption dominates in the first 5 min after the monomer is introduced, polymer deposition dominates thereafter. The formed layer exhibits poor adhesion to the surface as the PBS rinse removes the dissipative components of the film, leaving a highly compact film of PETE-S complexed with DOTAP that has a surface mass density of 1.1 μg/cm^2^. EIS data shows that the PETE-S layer produces an interface with higher impedance, which may be a result of poor contact of the PETE-S material with the gold electrode or the conversion of PETE-S into a nonconducting, dedoped state by the charged quaternary ammonium functional groups on DOTAP. However, the latter possibility can be eliminated based on the UV–vis data of polymerization in the solution presented in Figure S4, which shows that the presence of DOTAP has no effect on the broad polymer peak of PETE-S.

Comparing the behavior of the ETE-S monomer to that of the ETE-PC monomer, it is clear that the interactions of the ETE derivatives with the surface in both the monomer and the polymer form are controlled by the monomer side chain. While the ETE-PC monomer forms a thicker adsorbate layer on the DOTAP bilayer than on the bare gold layer, the adsorption is reversible and nondestructive, as evidenced by the return of the QCM-D frequency shift to the baseline after incubation of the bilayer with the ETE-PC monomer. On bare gold, the growth of the PETE-PC layer proceeds in two phases, where the initial deposition of a film similar in thickness to that observed for monomer adhesion is followed by the deposition of a thicker film once flow is stopped. Interestingly, this biphasic behavior is not observed on the DOTAP bilayer. EIS results show that the deposition of PETE-PC leads to a reduction in the impedance in the HRP-modified gold surface, indicating that the polymer forms an electrical contact with gold through the HRP layer. However, the magnitude of impedance reduction is less than that of the PETE-S on gold, partly due to the formation of a thinner polymer layer of 3.1 μg/cm^2^. Beyond surface–polymer interactions, the side chain affects the thermodynamics of both the oxidative polymerization and of the oxidation state of the resulting polymer. Namely, the redox potentials of both the monomer and the polymer shift to more positive values with an additional positive charge on the side chain.^13^ Thus, the higher impedance of the PETE-PC layer on gold relative to the PETE-S may also be partly caused by a change in the doping state of the PETE-PC polymer in addition to the difference in thickness. The shift of the monomer redox potential affects the kinetics of monomer oxidation by HRP, which likely affects the morphology of the resulting polymer layer, due to the dominance of the diffusion-controlled regime for ETE-S and the reaction-controlled regime for ETE-PC. As with ETE-S, when PETE-PC is deposited on the DOTAP bilayer, the impedance increases, which is likely due to poor contact with the gold surface.

Additionally, parameter values resulting from the circuit fitting analysis using modeling software across three sets of EIS measurements are listed in Table S2. The average polymer capacitances of Table S2 are normalized with respect to their corresponding specific mass values of Table S1, and the resulting mass specific capacitances are listed in Table S3. The estimated gravimetric capacitance values of 61 F/g for PETE-S on Au/HRP and 72 F/g for PETE-PC on Au/HRP are higher than the reported 20 F/g for in vivo polymerized PETE-S in xylem tissue^6,7^ but comparable to the reported 80–180 F/g range of poly(3,4-ethylenedioxythiophene) (PEDOT) specific capacitances.^32−34^

### Film Morphology

**Figure 5:**
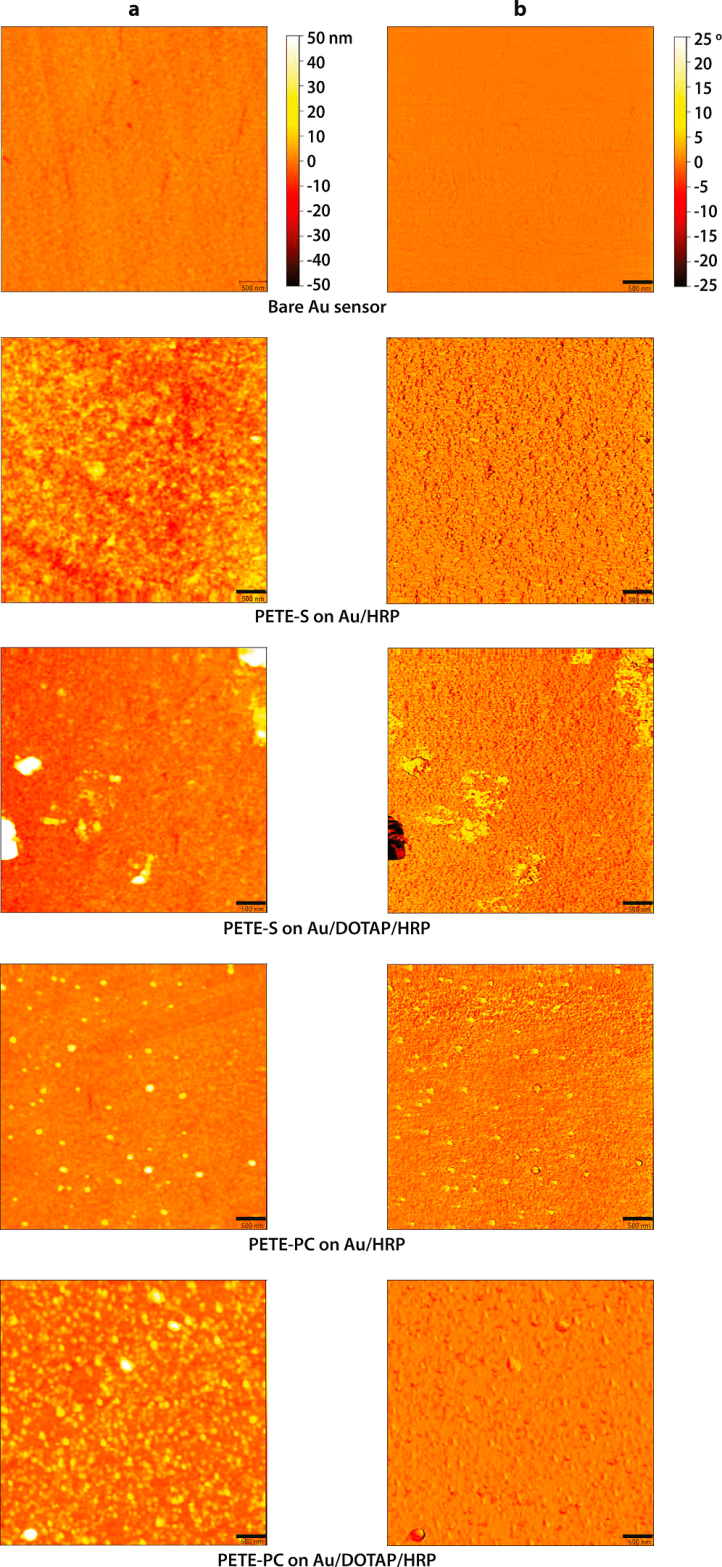
AFM topography at the central region of the five samples showing (a) height and (b) phase information, recorded for 5 μm × 5 μm scan size (or 512 × 512 pixels) and 500 nm scale bar.

The dry film morphology was characterized by AFM. All four samples measured by EQCM-D (PETE-S and PETE-PC with and without DOTAP bilayer) were investigated, along with a reference bare Au sensor. Five images, taken while focusing on the central region of the QCM sensor surface, are compiled in Figure 5. Corresponding RMS roughness values determined for the images are listed in Table 1.

**Table 1:** Roughness Value for Each (Entire) 5 μm × 5 μm Area Shown in Figure 5^a^

sample	rms roughness (nm)
bare Au	1.4
PETE-S on Au/HRP	5.5
PETE-S on Au/DOTAP/HRP	17.7
PETE-PC on Au/HRP	3.3
PETE-PC on Au/DOTAP/HRP	5.8

a Note that the roughness of the <10 nm height or <5° phase (i.e., nonyellow) regions of PETE-S on Au/DOTAP/HRP ranged from 2.2 to 2.4 nm while those of PETE-PC on Au/HRP ranged from 1.5 to 1.7 nm.

A tightly packed distribution of features was observed across the full image for PETE-S on Au/HRP and PETE-PC on Au/DOTAP/HRP. However, a relatively sparse and spread-out feature distribution was seen for PETE-PC on Au/HRP, resulting in a roughness value closest to that of the bare Au. PETE-S on Au/DOTAP/HRP had the highest roughness value among all the samples. Also, both PETE-S and PETE-PC had comparatively lower roughness values in the absence of the lipid bilayer.

To summarize, from the QCM-D measurements, the actual amount of the polymer seemed to depend on the distribution and availability of HRP active sites, the oxidation potential of the monomer, and the strength of the interactions between the monomer or polymer and the underlying substrate. Also, from the EIS data, it was inferred that the conductive polymer film was in contact with the Au electrode in the absence of a DOTAP lipid bilayer, whereas there was no contact between the polymer and the electrode surface when the bilayer was present. Furthermore, the postpolymerization PBS rinse caused minimal removal of polymer (mass) for the Au/HRP samples but significant loss of polymer for the Au/DOTAP/HRP samples. In particular, desorption of the polymer from Au/DOTAP/HRP during the PBS rinse was more pronounced for anionic PETE-S than for zwitterionic PETE-PC.

**Figure 6:**
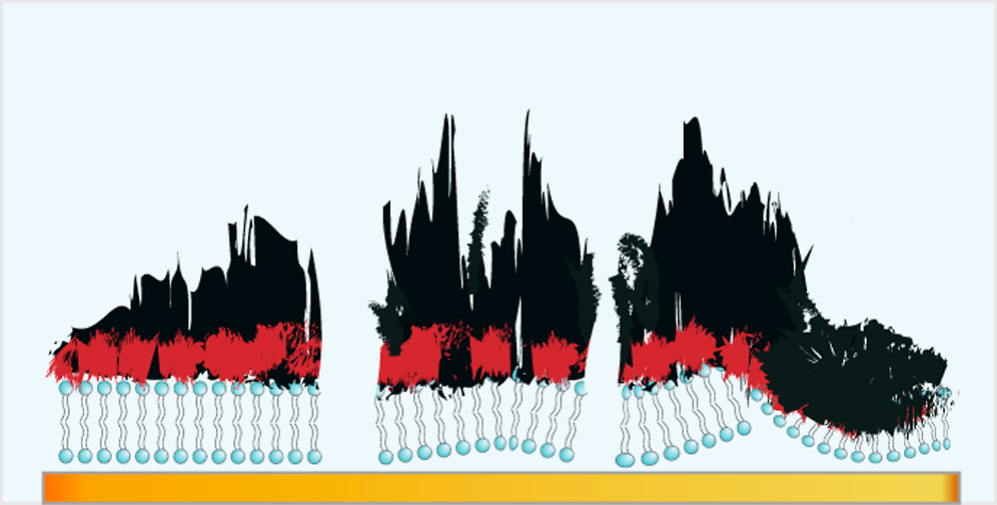
Updated schematic representation of the polymer on Au/DOTAP/HRP post-buffer rinse showing a partially disrupted DOTAP lipid bilayer along with polymer-lipid aggregation in a PBS medium.

These results, along with the corresponding AFM images of Figure 5, suggest the scenario illustrated in Figure 6, which is more complicated than the ideal polymer-on-bilayer representation of Figure 1, particularly for PETE-S on Au/DOTAP/HRP. Competition between the electrostatic interactions at the interface between the polymer and upper leaflet of the DOTAP bilayer, and the hydrophilic or hydrophobic interactions at the interface between the bilayer bottom leaflet and Au substrate, could disrupt the lipid bilayer at some places and result in the formation of lipid-polymer aggregates. These aggregates are then washed away by the PBS rinse, leaving behind regions of the Au electrode exposed to the electrolyte. In addition, some of these aggregates remain stuck to the Au electrode in the form of rough patches (Figure 5), contributing to an increase in the overall roughness (Table 1). This situation seems to be more pronounced for PETE-S on Au/DOTAP/HRP compared to PETE-PC on Au/DOTAP/HRP, which could be a consequence of the comparatively weaker interactions between the zwitterionic ETE-PC and the cationic DOTAP lipids, as opposed to the stronger interactions between the oppositely charged anionic PETE-S and the cationic lipid molecules. However, the final buffer rinse does not fully remove the materials adhered to the sensors, as evidenced by the nonzero frequency values recorded at the end of the measurement (Figure S1) and the AFM images (Figure 5). The surface charge of the monomer thus seems to play a key role, influencing not only the quantity of the polymer formed but also the quantity remaining attached to the substrate after the buffer rinsing.

## Conclusions

Simultaneous microgravimetric and impedance measurements are used to demonstrate the proof of concept for enzymatic in situ polymerization of ETE-S and ETE-PC on a model DOTAP lipid bilayer supported on a Au substrate in a PBS medium. The water-soluble monomers polymerize in the presence of H_2_O_2_ and HRP to form water-insoluble films that remain adhered to the underlying substrate, although to a larger extent on Au compared to the DOTAP lipid bilayer, after rinsing with buffer. The polymer-on-bilayer system presented in this work essentially serves as a simplified model to study the molecular interactions between enzymatically synthesized conductive polymers and lipid membranes before potential future in vivo applications. Estimated gravimetric capacitances and Young’s moduli of the resulting polymers on Au suggest that these two ETE-conjugates could be considered as suitable candidates for the development of implantable bioelectronic devices and subsequent implementation of in vivo therapeutics, with or without the involvement of Au interfaces. However, based on the system stability as evidenced by the extent of material removed during the final buffer rinse, PETE-PC seems to be slightly better suited for applications involving cationic lipids than the comparatively more disruptive PETE-S. These results expand the understanding, and thus the applicability, of this nascent field of *in situ*-formed bioelectronics. In the future, similar enzymatic polymerization studies of thiophene-based monomers on natural lipid extracts could be used as a rapid screen for binding and bilayer disruption before moving on to live cells or as a means of investigating the mechanism of polymer interactions with the live cell membrane.

## Supporting Information Available

QCM-D recordings of the four samples for the entire duration of the measurements, modeled parameters from QCM-D data for all measured overtones of the samples, impedance and phase spectra of various measurements, values of the fitted circuit elements obtained using modeling software, specific capacitance values for the polymers, overtone-normalized QCM-D recordings of additional control samples, absorbance spectra for all the samples used in QCM-D recordings, and topography images of additional samples (PDF)
