# Supplementary material for: Enzymatically Polymerized Organic Conductors on Model Lipid Membranes
Source: Langmuir. 2023 Jun 2;39(23):8196–204. doi: 10.1021/acs.langmuir.3c00654 (PMC10269430; doi:10.1021/acs.langmuir.3c00654)
Supplement: Supplementary file 1 — la3c00654_si_001.pdf [file la3c00654_si_001.pdf]

SUPPORTING INFORMATION

Enzymatically Polymerised Organic Conductors on Model Lipid Membranes

Diana Priyadarshini[1], Chiara Musumeci[1], David Bliman[2], Tobias Abrahamsson[1], Caroline Lindholm[1], Mikhail Vagin[1], Xenofon Strakosas[1], Roger Olsson[2,3], Magnus Berggren[1]*, Jennifer Gerasimov[1]*, Daniel T. Simon[1]

1. Laboratory of Organic Electronics, Department of Science and Technology, Linköping University, 601 74 Norrköping, Sweden.

2. Department of Chemistry and Molecular Biology, University of Gothenburg, Gothenburg 412 96, Sweden.

3. Chemical Biology and Therapeutics, Department of Experimental Medical Science, Lund University, Lund 221 84, Sweden.

Corresponding author: jennifer.gerasimov@liu.se, magnus.berggren@liu.se

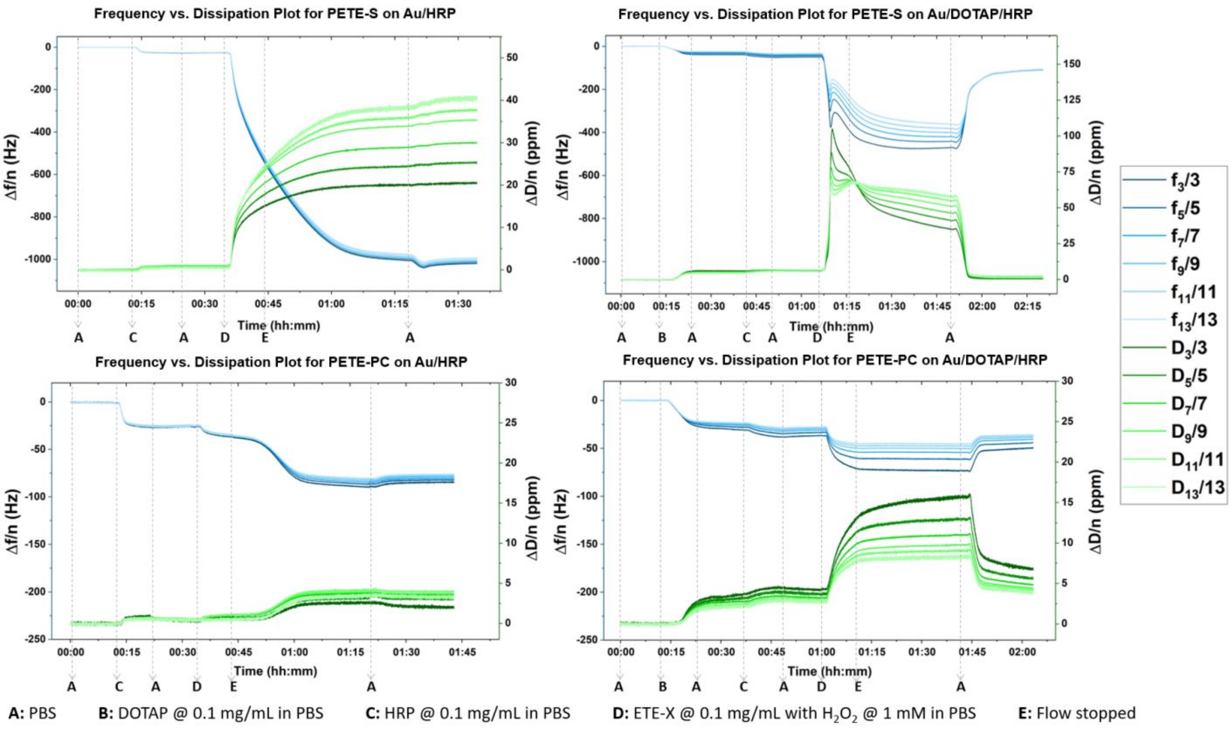

Figure S1: QCM-D recordings of the four samples for the entire duration of the measurements. Both the frequency and dissipation shifts are normalised with respect to their overtone numbers. Overtone spreading represents a thick film, while energy dissipation values approaching zero indicate a rigid film.[1,2]

Table S1: Modelled parameters from QCM-D data for all measured overtones of the samples. These values are estimated considering Kelvin-Voigt viscoelastic model for all cases except PETE-S on HRP/DOTAP where Saurbrey model is used since its ΔD values with respect to the bare Au surface are ~0.[1,2] In all cases, PBS rinse stage after HRP adsorption is fixed as the reference to model the fit for the corresponding polymer, and value at the end of the final PBS rinse is considered. Standard errors are calculated from four replicates. Elastic (or shear)[3] modulus and viscosity parameters are not estimated for PETE-S on Au/DOTAP/HRP since Saurbrey model assumes a rigid layer without any viscoelasticity. Comparatively higher error rates for shear modulus and viscosity values are probably due to their relatively higher sensitivity on the statistical deviation in the measured frequency and dissipation shifts caused by their coupled dependence on these parameters, as opposed to the linear dependence of Δf and ΔD on the modelled specific mass and thickness values.[4]

**Sample**	**Thickness (nm)**	**Specific mass (μg/cm²)**	**Viscosity (mPa·s)**	**Shear modulus (KPa)**
PETE-S on Au/HRP	167.7 ± 15.4	16.5 ± 1.5	51.2 ± 11.8	1873.7 ± 402.7
PETE-S on Au/DOTAP/HRP	10.8 ± 4.6	1.1 ± 0.4	–	–
PETE-PC on Au/HRP	31.3 ± 8.6	3.1 ± 0.8	3.8 ± 0.7	358.6 ± 313.7
PETE-PC on Au/DOTAP/HRP	14.2 ± 10.4	1.4 ± 1.0	1.4 ± 0.1	71.3 ± 28.0

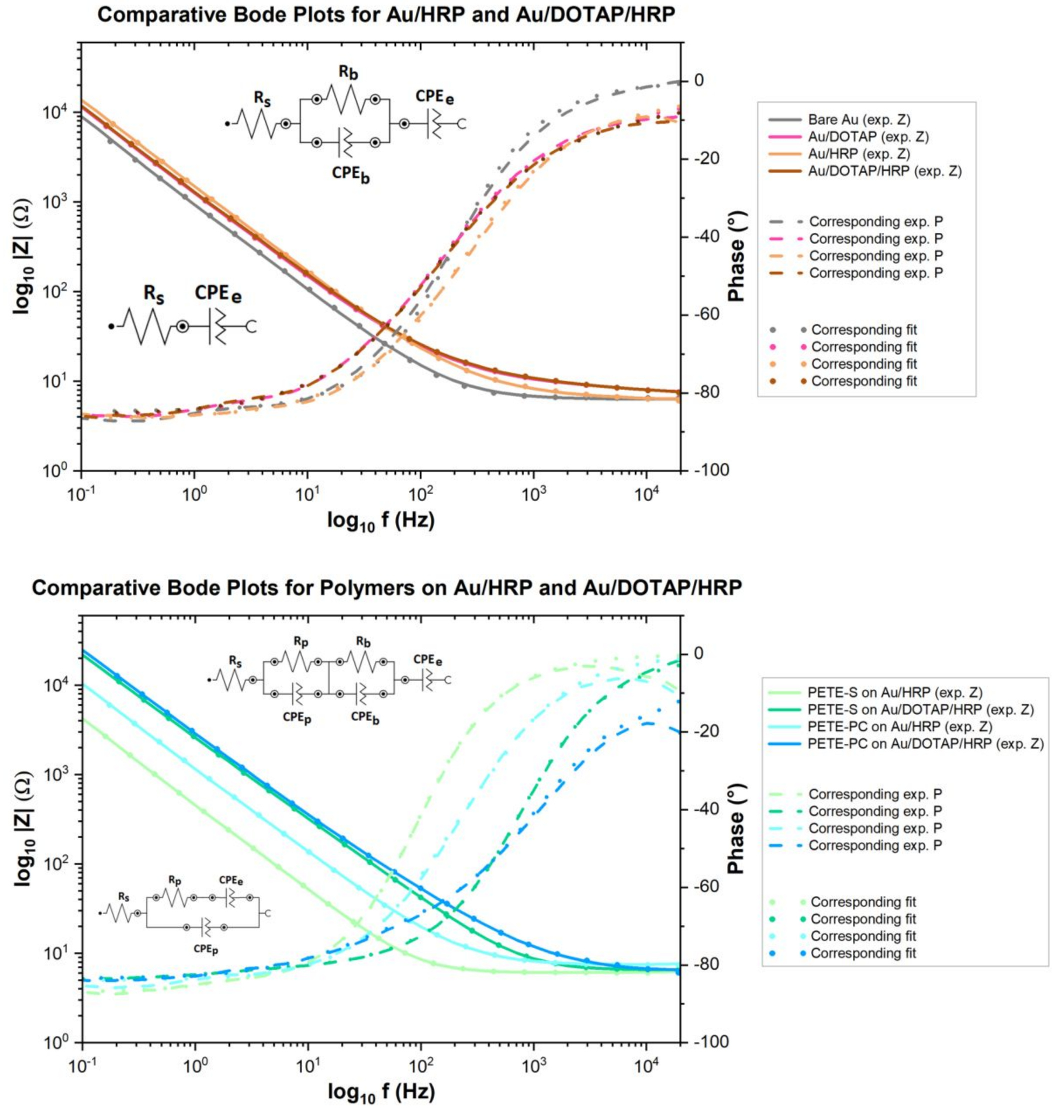

Figure S2: Impedance and phase spectra of various measurements. Dotted plots represent the fit data which are modelled from the inset circuits, with the bottom-left circuit representing bare Au or PETE-X on Au/HRP, while the circuit on the top represents Au/HRP or Au/DOTAP or Au/DOTAP/HRP or PETE-X on Au/DOTAP/HRP. Quantitative analysis of EIS data is performed using these inset circuits. A simple circuit consisting of solution resistance connected in series with electrode capacitance is used to represent the bare Au system, while the circuit containing a parallel resistance-capacitance branch connected in between these two elements is used to represent the impedance contribution of the DOTAP bilayer or HRP or HRP on DOTAP layer.[5] Constant phase element (CPE) is used instead of an ideal capacitor for a better fit, considering the inhomogeneities of the layers and interfaces.[6] The capacitance of this element is $\boldsymbol{C} = \left(\boldsymbol{P}(\boldsymbol{R_s})^{(\mathbf{1}-\boldsymbol{\phi})}\right)^{\mathbf{1}-\boldsymbol{\phi}}$, where R_s is the solution resistance, P is the fitting parameter, and ϕ is an exponent factor ranging from 0 for pure resistance to 1 for pure capacitance.[6,7] Based on the trends observed in the Bode plots (Figure 4 of the main paper), polymers on Au/HRP are modelled using an equivalent circuit developed to represent an electrode with an organic protective coating,[7–10] while polymers on Au/DOTAP/HRP are modelled with two parallel RC units between the PBS electrolyte and the Au electrode. . Equivalent circuits used to model the system impedance response consists of electrolyte solution resistance R_s and electrode capacitance CPE_e, along with R_p and CPE_p which respectively represent the resistance and capacitance of the polymer, while R_b and CPE_b represent the bilayer resistance and capacitance respectively.

Table S2: Values of the fitted circuit elements obtained using modelling software for frequencies ranging from 0.1 Hz to 20 kHz of the input AC signal. Capacitances of CPEs are calculated using the equation in the Figure S2 caption. Standard errors are from at least three replicates. Considering the low ϕ values (much lower than the ideal 1) of several CPE elements, the resulting capacitances hereby calculated using the previously specified equation should be considered as estimates only.[7]

Sample	R_s (Ω)	R_p (Ω)	CPE_p		C_p (μF)	$R_{b/h}$ (Ω)	$CPE_{b/h}$		$C_{b/h}$ (μF)	CPE_e		C_e (μF)
			P_p x10^{-3} ($\Omega^{-1}s^{\phi}$)	ϕ_p			$P_{b/h}$ x10^{-3} ($\Omega^{-1}s^{\phi}$)	ϕ_b		P_e x10^{-3} ($\Omega^{-1}s^{\phi}$)	ϕ_e	
Bare Au	6.0 ± 0.2	-	-	-	-	-	-	-	-	0.2 ± 0.0	0.9 ± 0.0	70.9 ± 11.3
Au/HRP	5.4 ± 0.4	-	-	-	-	182.0 ± 47.1	7.8 ± 4.3	0.5 ± 0.1	81.7 ± 32.3	0.1 ± 0.0	1.0 ± 0.0	64.3 ± 6.0
Au/DOTAP	5.3 ± 0.7	-	-	-	-	328.5 ± 39.4	7.5 ± 4.3	0.5 ± 0.1	24.2 ± 10.8	0.1 ± 0.0	1.0 ± 0.0	104.5 ± 12.2
Au/DOTAP/HRP	4.9 ± 0.7	-	-	-	-	341.1 ± 71.9	7.3 ± 4.3	0.5 ± 0.1	16.7 ± 7.3	0.1 ± 0.0	1.0 ± 0.0	101.1 ± 12.0
PETE-S on Au/HRP	6.1 ± 0.2	1867.6 ± 634.4	0.3 ± 0.1	0.9 ± 0.0	214.5 ± 47.6	-	-	-	-	0.1 ± 0.0	1.0 ± 0.0	60.7 ± 21.0
PETE-S on Au/DOTAP/HRP	5.2 ± 0.8	12.7 ± 9.2	0.5 ± 0.3	0.9 ± 0.1	223.1 ± 164.5	95.0 ± 28.9	0.6 ± 0.2	0.9 ± 0.0	355.6 ± 121.8	0.1 ± 0.0	0.9 ± 0.0	39.9 ± 3.6
PETE-PC on Au/HRP	6.3 ± 0.6	217.1 ± 186.4	0.1 ± 0.0	0.9 ± 0.0	38.0 ± 16.9	-	-	-	-	0.0 ± 0.0	0.9 ± 0.0	15.8 ± 3.4
PETE-PC on Au/DOTAP/HRP	4.8 ± 0.2	5.0 ± 3.6	0.8 ± 0.7	0.8 ± 0.2	15.8 ± 3.8	158.6 ± 79.1	0.4 ± 0.1	0.7 ± 0.0	50.1 ± 21.0	0.1 ± 0.0	0.9 ± 0.0	48.8 ± 17.0

Table S3: Specific capacitance values for the polymers calculated from the corresponding modelled capacitance of Table S2 and specific mass of Table S1, considering 5 mm diameter active sensing spot[11] in the calculations. Errors correspond to standard errors calculated across three EIS datasets. Note that the respective gravimetric capacitances for PETE-x on Au/DOTAP/HRP are calculated with the assumption of separate uniform layers *i.e.*, polymer-on-bilayer stacked on top of each other on a Au electrode. Since the actual scenario might be different (as represented in Figure 6 of the main paper), these particular polymer-on-bilayer capacitance values might represent mathematical estimations but without any practical significance as such.

Sample	Mass Specific Capacitance (F/g)
PETE-S on Au/HRP	61.0 ± 15.5
PETE-S on Au/DOTAP/HRP	2449.9 ± 1685.8
PETE-PC on Au/HRP	71.8 ± 13.9
PETE-PC on Au/DOTAP/HRP	216.7 ± 61.3

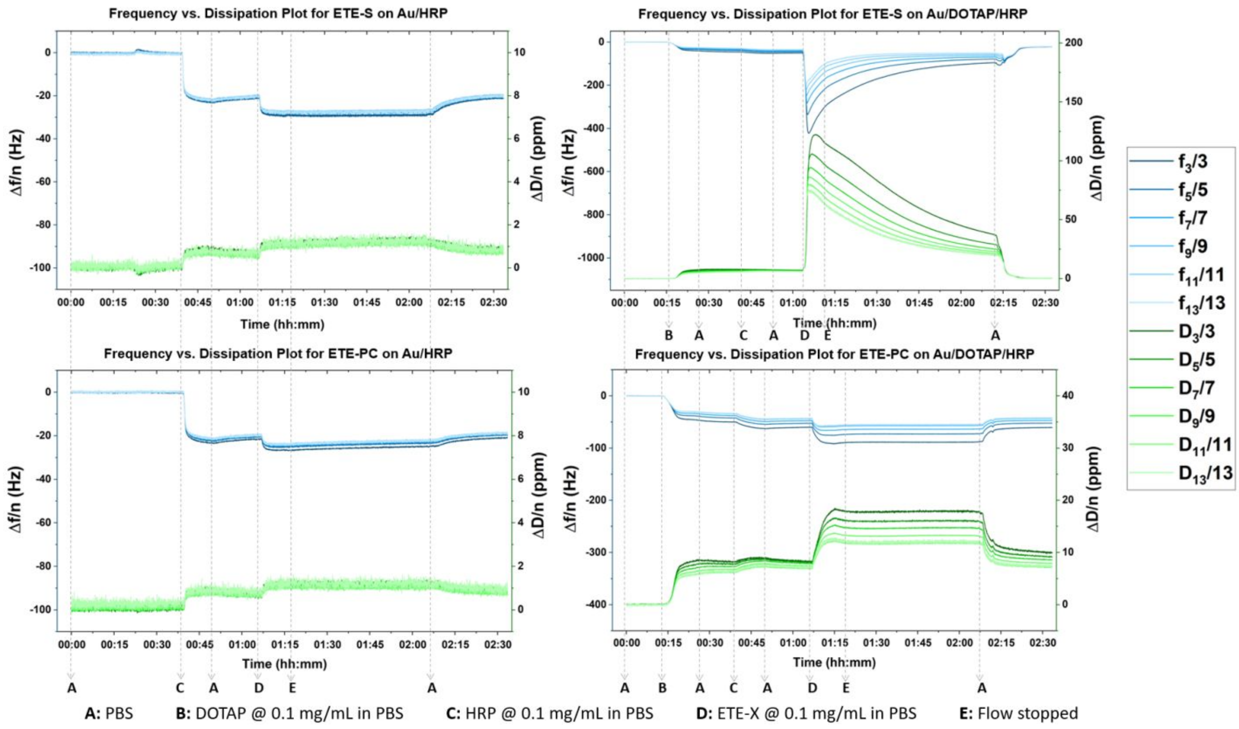

Figure S3: Overtone-normalised QCM-D recordings of additional control samples for the entire duration of the measurements. Monomer is deposited on HRP in the absence of H_2O_2 to prevent enzymatic polymerisation. Consequently, most of the monomer is washed off during the final PBS rinse stage since it remains water-soluble due to lack of polymerisation. Prominent dip in the frequencies at time 1:05 for the case of ETE-S on Au/DOTAP/HRP is probably due to the stronger electrostatic attraction between the oppositely charged monomer and cationic lipids of the bilayer, compared to the zwitterionic ETE-PC molecules.

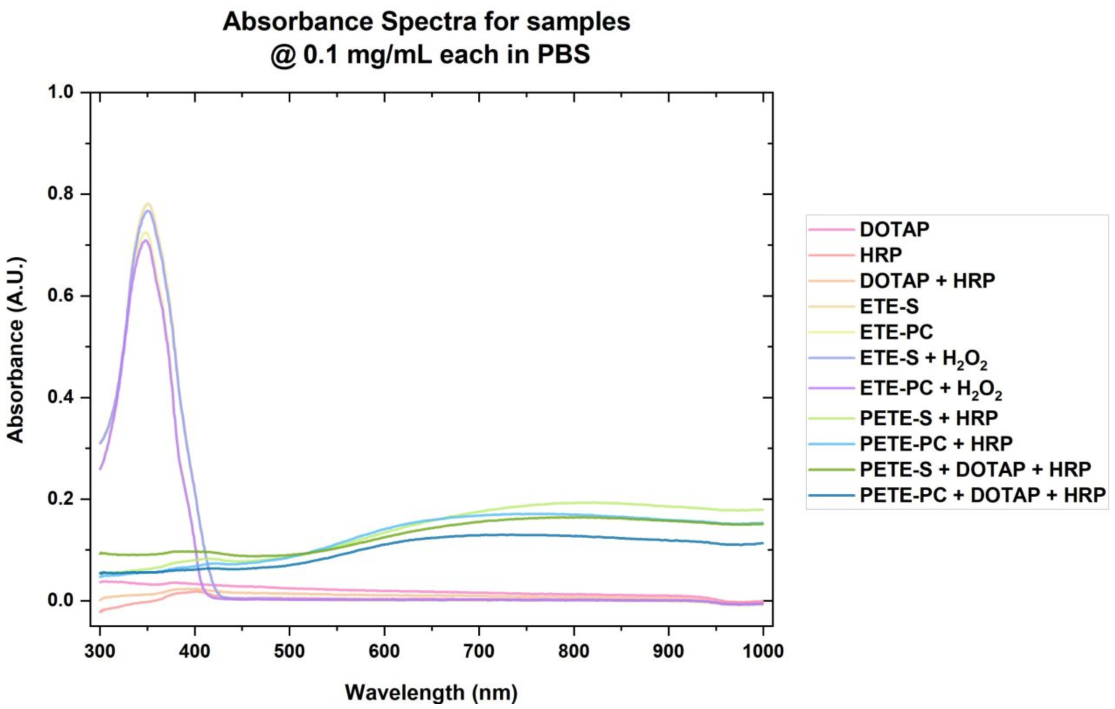

Figure S4: Absorbance spectra for all the samples used in QCM-D recordings, measured using microplate reader. Absorbance values are averaged from triplicates for each sample, and absorbance of reference PBS sample is subtracted from the sample absorbance values to exclude its contribution. Monomer peaks centred at 350 nm disappear when ETE-S and ETE-PC are enzymatically polymerised by HRP in the presence of H_2O_2 oxidiser. Comparing with similar spectra in published reports,[12–14] the absence of monomer peaks at 350 nm along with the presence of broad peaks starting at ~500 nm, confirm that both ETE-S and ETE-PC are fully polymerised, and that resulting polymers PETE-S and PETE-PC are in their doped state, both in the presence and absence of DOTAP lipids.

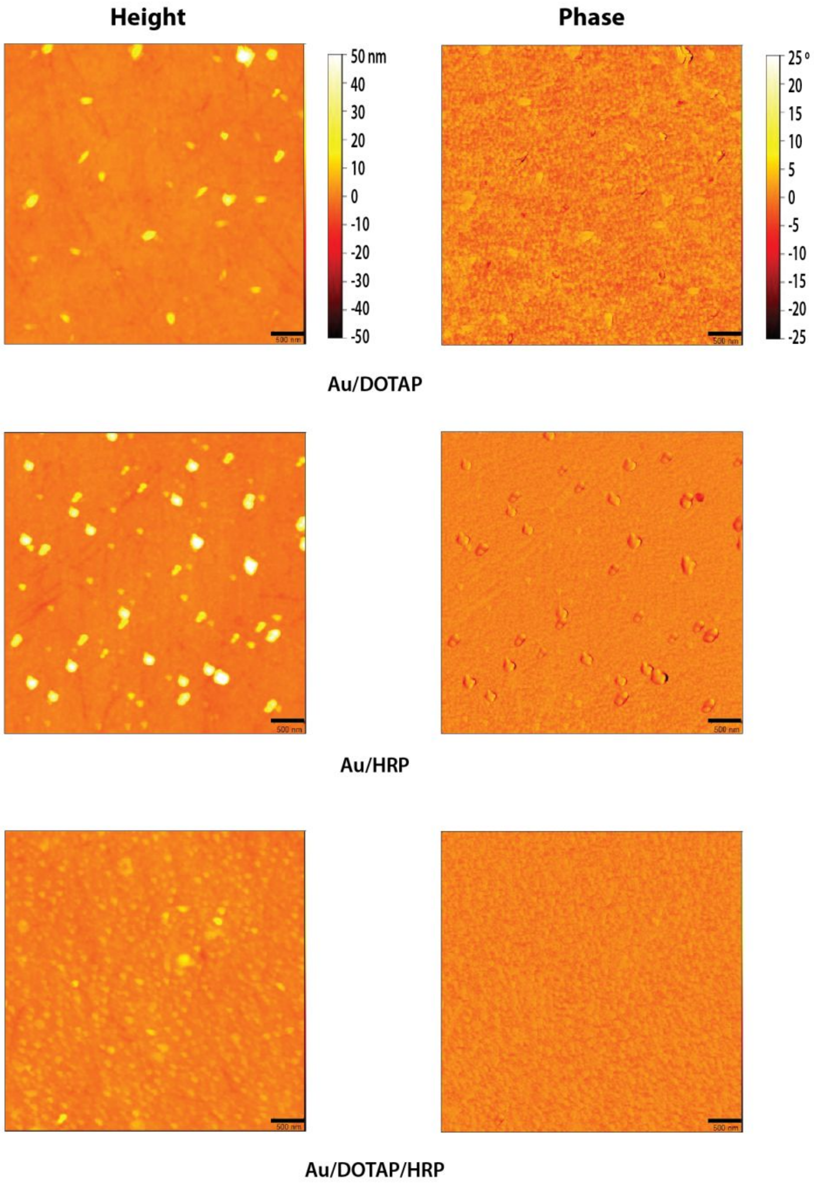

Figure S5: Topography images of additional samples indicating height and phase information. The samples were imaged over a scan size of 5 μm^2 (or 512 × 512 pixels), and the scale bar is of 500 nm. Corresponding colour bars of the images are indicated on the right. Estimated RMS roughness values of these images obtained using Gwyddion software are 3.1 nm, 6.2 nm, and 1.9 nm, respectively.

References

(1) McCubbin, G. A.; Praporski, S.; Piantavigna, S.; Knappe, D.; Hoffmann, R.; Bowie, J. H.; Separovic, F.; Martin, L. L. QCM-D Fingerprinting of Membrane-Active Peptides. *Eur. Biophys. J.* **2011**, *40* (4), 437–446. https://doi.org/10.1007/S00249-010-0652-5.

(2) Lu, N. Y.; Yang, K.; Li, J. L.; Yuan, B.; Ma, Y. Q. Vesicle Deposition and Subsequent Membrane–Melittin Interactions on Different Substrates: A QCM-D Experiment. *Biochim. Biophys. Acta - Biomembr.* **2013**, *1828* (8), 1918–1925. https://doi.org/10.1016/J.BBAMEM.2013.04.013.

(3) McNamara, T. P.; Blanford, C. F. A Sensitivity Metric and Software to Guide the Analysis of Soft Films Measured by a Quartz Crystal Microbalance. *Analyst* **2016**, *141* (10), 2911–2919. https://doi.org/10.1039/C6AN00143B.

(4) Parveen, N.; Jana, P. K.; Schönhoff, M. Viscoelastic Properties of Polyelectrolyte Multilayers Swollen with Ionic Liquid Solutions. *Polym. 2019, Vol. 11, Page 1285* **2019**, *11* (8), 1285. https://doi.org/10.3390/POLYM11081285.

(5) Nissa, J.; Janson, P.; Berggren, M.; Simon, D. T. The Role of Relative Capacitances in Impedance Sensing with Organic Electrochemical Transistors. *Adv. Electron. Mater.* **2021**, *7* (4), 1–9. https://doi.org/10.1002/aelm.202001173.

(6) Briand, E.; Zäch, M.; Svedhem, S.; Kasemo, B.; Petronis, S. Combined QCM-D and EIS Study of Supported Lipid Bilayer Formation and Interaction with Pore-Forming Peptides. *Analyst* **2010**, *135* (2), 343–350. https://doi.org/10.1039/B918288H.

(7) Musumeci, C.; Vagin, M.; Zeglio, E.; Ouyang, L.; Gabrielsson, R.; Inganäs, O. Organic Electrochemical Transistors from Supramolecular Complexes of Conjugated Polyelectrolyte PEDOTS. *J. Mater. Chem. C* **2019**, *7* (10), 2987–2993. https://doi.org/10.1039/C8TC05774E.

(8) Loveday, D.; Peterson, P.; Rodgers-Gamry Instruments, B. EXPOSURE TESTS Evaluation of Organic Coatings with Electrochemical Impedance Spectroscopy Part 3: Protocols for Testing Coatings with EIS EIS and Atmospheric Exposure Tests Immersion and Measurement of Impedance Magnitude at 0.1 Hz. *JCT CoatingsTech* **2005**, *February*, 22–27.

(9) O'Donoghue, Mike; Garrett, R.; Datta, V.; Roberts, P.; Aben, T. Electrochemical Impedance Spectroscopy: Testing Coatings for Rapid Immersion Service. *Mater. Perform.* **2003**, *42* (9), 36–41.

(10) Waters, N.; Connolly, R.; Brown, D.; Laskowski, B. Electrochemical Impedance Spectroscopy for Coating Evaluation Using a Micro Sensor. *Annu. Conf. PHM Soc.* **2014**, *6* (1), 2340.

(11) Richter, R. P.; Rodenhausen, K. B.; Eisele, N. B.; Schubert, M. Coupling Spectroscopic Ellipsometry and Quartz Crystal Microbalance to Study Organic Films at the Solid–Liquid Interface. *Springer Ser. Surf. Sci.* **2018**, *52*, 391–417. https://doi.org/10.1007/978-3-319-75895-4_17.

(12) Dufil, G.; Parker, D.; Gerasimov, J. Y.; Nguyen, T. Q.; Berggren, M.; Stavrinidou, E. Enzyme-Assistedin Vivopolymerisation of Conjugated Oligomer Based Conductors. *J. Mater. Chem. B* **2020**, *8* (19), 4221–4227. https://doi.org/10.1039/d0tb00212g.

(13) Mantione, D.; Stavrinidou, E.; Pavlopoulou, E.; Istif, E.; Dufil, G.; Vallan, L.; Parker, D.; Brochon, C.; Cloutet, E.; Hadziioannou, G.; Berggren, M. Thiophene-Based Trimers for in Vivo Electronic Functionalization of Tissues. *ACS Appl. Electron. Mater.* **2020**, *2* (12), 4065–4071. https://doi.org/10.1021/acsaelm.0c00861.

(14) Volkov, A. V.; Singh, S. K.; Stavrinidou, E.; Gabrielsson, R.; Franco-Gonzalez, J. F.; Cruce, A.; Chen, W. M.; Simon, D. T.; Berggren, M.; Zozoulenko, I. V. Spectroelectrochemistry and Nature of Charge Carriers in Self-Doped Conducting Polymer. *Advanced Electronic Materials*. **2017**, 3, 1700096. https://doi.org/10.1002/aelm.201700096.
